# Openness to experience, a personality trait that reduces susceptibility to memory age-based stereotype threat

**DOI:** 10.3389/fpsyg.2024.1399131

**Published:** 2024-07-23

**Authors:** Badiâa Bouazzaoui, Séverine Fay, Emilie Alibran, Léa Martinez, Florent Pinard, Nolwenn Kerhardy, Tugba Onsekiz, Laurence Taconnat

**Affiliations:** UMR CNRS 7295, Centre de Recherches sur la Cognition et l’Apprentissage, Université de Tours, Université de Poitiers, Tours, France

**Keywords:** stereotype threat, aging, episodic memory, strategy, openness trait personality

## Abstract

**Introduction:**

Age-based stereotype threat (ABST), the concern of being judged according to a negative age stereotype may lead to underperformance in the stereotype domain. The present study aims to replicate the negative effect of ABST on episodic memory. Importantly, we further examine openness to experience as a potential buffer of the ABST effect as well as the role that different memory strategies may play in episodic memory performance.

**Method:**

Seventy-five older adults were randomly assigned to the ABST condition or the control condition before taking a word-stem cued recall memory task. They learned word-lists with either a repetition strategy, low resource demanding but less efficient, or a mental imagery strategy, high resource demanding but more efficient. Openness was measured with the Big-5 personality questionnaire.

**Results:**

ABST reduced memory performance and disrupted more the recall of words learned with the imagery strategy. The results also showed that openness predicted recall performance associated with the imagery strategy only in the threatened group.

**Conclusion:**

These results indicated that a high level in openness may disrupt the negative effect of ABST by improving the capacity of threatened people to execute efficient, resource demanding memory strategies. This finding supports the idea that contextual factors as well as individual characteristics such as personality, need to be considered when assessing episodic memory in aging.

## 1 Introduction

A socially shared stereotype is that getting old means having a failing memory. Stereotypes can lead to the phenomenon of stereotype threat (Steele and Aronson, 1995), defined as the risk of applying a negative stereotype about one’s group to oneself. The negative impact of stereotype on performance has been studied through different fields such as racial, gender and age differences (review, Barber, 2017). There is now an abundant literature on age-based stereotype threat (ABST), especially with regard to memory. In these studies, older adults were subliminally primed with aging stereotype cues (Levy, 1996; Stein et al., 2002; Bouazzaoui et al., 2016, 2020; Lemaire et al., 2018) and/or an instruction manipulation in which the memory component of the task was emphasized (Rahhal et al., 2001; Stein et al., 2002; Hess et al., 2003, 2009; Desrichard and Kopetz, 2005; Hess, 2005; Hess and Hinson, 2006; Mazerolle et al., 2012; Bouazzaoui et al., 2016, 2020; Lemaire et al., 2018; for meta-analyses see Armstrong et al., 2017 and Lamont et al., 2015). A well-replicated finding is that older adults underperformed when they were stereotyped as being less efficient in performing a memory task. Stereotype threat not only affects performance on laboratory tasks, but also clinical assessment for predementia (Mazerolle et al., 2017). Thus, ABST may lead to overestimating the magnitude of the age-related effect on memory.

Studies examining the processes underlying the stereotype threat effect have identified several factors as determinants or mediators, supporting a multifactorial hypothesis (see Barber, 2017). Among these factors, a possible key variable is the ability to implement controlled processes when faced with stereotype threat. According to Schmader et al. (2008), these processes are difficult to implement when cognitive resources are depleted by coping with intrusive thoughts and the anxiety they produce. Mazerolle et al. (2012) confirmed that stereotype threat interferes with the working memory of older adults, reducing controlled processes and increasing automatic processes. It is now well-known that controlled processes support the implementation of efficient memory strategies (Bryan et al., 1999; Taconnat et al., 2006, 2007; Bouazzaoui et al., 2010; Burger et al., 2017; and see Lemaire, 2010 for a review). Therefore, one might reasonably expect that by reducing the ability to implement controlled processes, ABST would also affect the use of efficient strategies.

Efficient strategies are needed to optimize encoding and retrieval processes. Because older adults generally have reduced cognitive resources, they fail to initiate efficient strategies adopted spontaneously by younger adults, for example, increasing study time to cope with task difficulty (Murphy et al., 1981; Souchay and Isingrini, 2004; Froger et al., 2012) or using an imagery strategy (Froger et al., 2012). Hess et al. (2003) found that the effect of ABST on memory performance was mediated by strategy use, participants making less use of semantic clustering as a recall strategy when under threat. More recently, Lemaire et al. (2018) examined the ABST effect on the ability to select and execute two memory strategies, one low resource demanding (repetition), the other high resource demanding but more efficient (imagery) as it allows deeper encoding and leads to better memory performance (Craik and Lockhart, 1972; Paivio and Csapo, 1973; Froger et al., 2012; Hertzog et al., 2012; Burger et al., 2017). Lemaire et al. (2018) showed that ABST affected mainly the selection and execution of the most resource demanding strategy (i.e., imagery). Examining the level of processing at encoding through imagery and repetition strategies sheds light on the underlying mechanisms of the effect of ABST on episodic memory. The present study aimed to extend this finding with a new direction that was to investigate the role of personality in susceptibility to ABST. The variability observed in older adults’ memory performance might be explained in part by personality and although several personality traits may be associated with cognitive performance, the main one seems to be openness to experience (Simon et al., 2020), defined as the tendency to be intellectually curious, creative, unconventional and imaginative. In this study we were particularly interested in this personality trait because it appears to support both episodic memory performance and, more specifically, the implementation of strategies that are altered by ABST. The present study was then designed to explore a new and interesting topic, namely how open people might deal with ABST and whether openness might provide some protection against the negative effects of ABST on the execution of effortful encoding strategies.

Openness is positively correlated with engagement in a variety of cognitively stimulating activities (Kraaykamp and van Eijck, 2005) and also to high cognitive performance including episodic memory (e.g., Soubelet and Salthouse, 2010; Simon et al., 2020; Karsazi et al., 2021). Studies examining the role of openness in older adults have also demonstrated a beneficial effect of openness on episodic memory (Martinez et al., 2021; Taconnat et al., 2022a). In addition to supporting memory functioning, openness also appears to be a factor of cognitive reserve protecting against the effects of age (Franchow et al., 2013; Luchetti et al., 2016; Jackson et al., 2020; Simon et al., 2020; Karsazi et al., 2021). Some studies have also hypothesized that the beneficial effect of openness on performance may depend on the experimental context. Using the generation effect paradigm (Martinez et al., 2021; Taconnat et al., 2022a) or the prior task success paradigm (Taconnat et al., 2022a) to vary the level of difficulty of the task in terms of the cognitive resources required, the authors found that openness helps to improve memory performance mainly when the task is resource-dependent. This beneficial effect can be explained by the fact that openness leads to cognitive stimulation, thereby facilitating the acquisition of crystallized knowledge and fluid intelligence, which are therefore less affected by age (Salthouse et al., 2002; Moutafi et al., 2006; Jopp and Hertzog, 2007; Zimprich et al., 2009), and are essential resources for memory functioning (Bouazzaoui et al., 2013, 2014; Guerrero-Sastoque et al., 2020). Considering the relationship between openness and the implementation of memory strategies, Talpain and Soubelet (2020) observed that openness was positively associated with the use of strategies supporting memory performance, and that strategy use mediated the positive effect of openness on episodic memory functioning. This mediation effect was more significant when the strategy involved imagery rather than repetition, indicating that openness mainly affects the most resource-dependent memory strategy.

Thus, in contrast to ABST, openness to experience positively supports memory performance mainly in effortful cognitive tasks, due in part to the exposure to cognitive-stimulating life experiences which increase the ability to implement efficient strategies. To examine the interaction between ABST and openness in older adults’ episodic memory performance, the present study took a dual approach, extending findings that ABST has a negative impact on the use of memory strategies (Hess et al., 2003; Lemaire et al., 2018), and those that observed a beneficial effect of openness on memory performance and strategic abilities (Talpain and Soubelet, 2020; Martinez et al., 2021; Taconnat et al., 2022a). Older adults were randomly assigned to the ABST condition or the control condition before carrying out a word-stem cued-recall memory task. Two strategies were proposed at encoding: repetition for half of the words and mental imagery for the other half. Openness was measured using the Big Five Inventory (John et al., 1991; Plaisant et al., 2010). In line with Lemaire et al. (2018), given that stereotype threat consumes attentional resources and that the imagery strategy is highly resource demanding, we first expected that ABST would reduce memory performance, and more so in the imagery condition than in the repetition condition. Second, we expected stereotype threat to interact with strategy and openness. If stereotype endorsement depends on personality, we can expect that individuals with higher levels of openness would be less susceptible to stereotype threat than those who are less open to experience. Furthermore, if openness supports memory performance in the more resource-dependent situations, it would be mainly related to memory performance in the threatened group by counteracting the negative effect of stereotype threat and hence improving the ability to execute more effortful but also more efficient strategies.

## 2 Method

### 2.1 Participants

The size of the target sample was determined using an *a priori* power analysis using R software with the pwr.f2.test function (R Core Team, 2021). In line with Lamont et al. (2015) and previous studies using a similar experimental design, we used a d of 0.52. Our mixed design could achieve 80% power with a total of 59 participants. We decided to exceed this criterion and recruit 80 participants, 40 per group to anticipate the risk of having to exclude participants. Finally, the preliminary analyses have led to the exclusion of 5 participants, one in the control condition and four in the threatened condition because their MMSE score was less than 27 (see below).

The sample consisted of 75 native French speakers aged between 60 and 78, recruited from the general community by advertising and word of mouth. They were randomly assigned to either the control (*n* = 39) or the threatened condition (*n* = 36). The characteristics of participants in each group are summarized in Table 1. The groups did not differ significantly in age, educational level, proportion of females, vocabulary (Mill Hill vocabulary test, Deltour, 1993) or in openness to experience (John et al., 1991; Plaisant et al., 2010). None of the participants had a history of brain injury, cardiovascular disease, psychiatric disease or alcoholism, and all had normal or corrected-to-normal vision. None of them were taking medication known to affect the central nervous system. All the participants scored below the cut-off of 11 on the two subscales of the Hospital Anxiety and Depression Scale (HADS, Zigmond and Snaith, 1983), with no significant difference between groups. They scored at or above the cut-off of 27 on the Mini-Mental State Examination (MMSE, Folstein et al., 1975), suggesting that they were not suffering from cognitive impairment. The study was approved by the local ethics committee of the University of Tours, and all participants were volunteers and signed consent forms.

**Table 1:** Characteristics of participants per group (means and standard deviations).

	Control group (*n* = 39)	Threatened group (*n* = 36)	*t* (73)	*p*
	M (SD) Range	M (SD) Range		
Age	68.05 (4.90) [60–78]	68.08 (4.63) [60–78]	−0.02	0.97
Percentage of females	54	69	χ2 (1) = 1.92	0.16
Education level	11.64 (2.52) [8–17]	12.36 (1.95) [8–16]	−1.37	0.17
Vocabulary (Mill Hill)	26. 90 (4.50) [15–33]	26.83 (3.30) [20–31]	0.06	0.94
Anxiety (HADS)	5.69 (2.22) [2–10]	5.30 (2.64) [2–10]	0.68	0.49
Depression (HADS)	5.74 (2.07) [2–10]	5.08 (2.83) [2–10]	1.15	0.25
Openness	31.25 (5.19) [18–42]	31.75 (7.06) [20–43]	−0.34	0.72

HADS, Hospital Anxiety and Depression Scale.

### 2.2 Material and procedure

During an initial meeting with the experimenter, participants completed the screening measures (Mill Hill vocabulary test, HADS, MMSE). They also completed the French version of the Big Five Inventory (John et al., 1991; Plaisant et al., 2010). This self-report questionnaire rates participants on five personality traits (Cronbach’s alpha = 0.74): Extraversion, Agreeableness, Conscientiousness, Neuroticism and Openness. For the purposes of the present study, we analyzed only the scores for “Openness to experience,” which has 10 items rated on a five-point Likert scale, from “Strongly disapprove” to “Strongly approve.” A high score indicates a high level of openness to experience.

In the second session, as previous studies (Bouazzaoui et al., 2016; Lemaire et al., 2018). Participants in the threatened group were primed with a questionnaire to activate the aging stereotype. The material, inspired by previous studies (Levy, 1996; Stein et al., 2002), consisted of 14 adjectives describing older people, 7 positive and 7 negative. Participants had to indicate orally the degree to which each adjective was relevant to an older adult, from “not at all relevant” to “very relevant.” Next, all the participants performed the episodic memory task. The stereotype threat was manipulated through the information given to the participants (Bouazzaoui et al., 2016, 2020; Lemaire et al., 2018). For the threatened group, the memory component of the task was emphasized as follows: “You are participating in a study on the effects of aging on memory. More specifically, we want to know whether, as shown by other studies in psychology, memory performance tends to decline with age. We will compare your memory performance to that of a group of young adults.” For the control group, the instructions deemphasized the memory component of the test: “You are participating in a study on vocabulary knowledge. This is why you are now going to carry out tasks based on vocabulary understanding.”

Participants performed a word-stem cued-recall task comprising six study-test blocks (Angel et al., 2010; Guerrero et al., 2022). The material consisted of 270 words divided into six lists (one list per block) of 45 words, matched for mean number of letters and mean frequency of occurrence.

**Figure 1:**
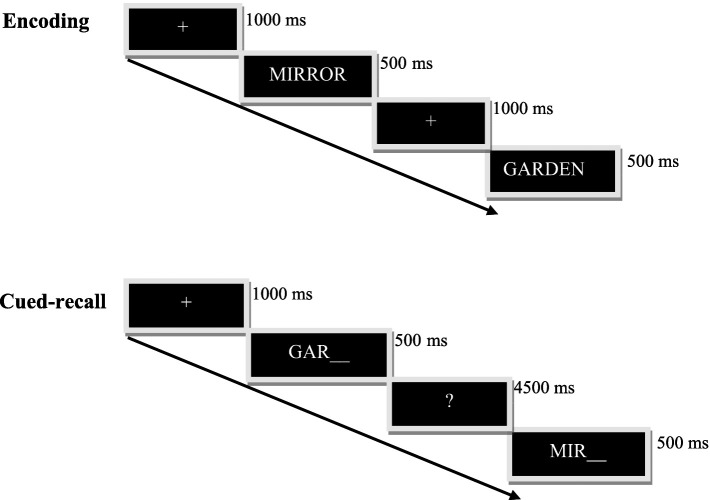
Visual depiction of the sequence of events in the encoding and cued-recall memory phases.

In the encoding phase (see Figure 1) 30 items were presented one by one in uppercase for 500 msec. Participants were told that they would see words on the screen that they would have to learn. For half the blocks, they were told they could repeat the words as many times as they liked. For the other half, they were invited to create a mental image of the words. The order of the strategies was counterbalanced across blocks; half the participants started with the imagery strategy and the other half started with the repetition strategy. We chose to use three blocks per condition to obtain a large number of events per strategy condition and per group and therefore a sensitive memory measure.

Each cued-recall phase (see Figure 1) comprised 45 word-stems displayed one by one for 500 msec, 30 from the study list and 15 not presented during the encoding phase. These word stems consist of the three first letters of a word and were all different (i.e., the words chosen to make the word lists all begin with different three-letter stems). Participants were instructed to complete each stem with a studied word, or failing that, with another suitable word, and then indicating whether the completion corresponded to a studied (old) or an unstudied (new) word. Participants had 4.5 s to answer orally.

At the beginning of each of the six experimental blocks, the instructions manipulating the stereotype threat were repeated in order to maintain the effect. The six word lists were counterbalanced across strategy conditions and across participants, the sublist used for distractors was counterbalanced across participants, and the words in each list were presented in random order. Before starting the test phase, participants first completed a familiarization phase to understand the instructions and the procedure. During this phase, they encoded a list of 6 words using the repetition strategy followed by a cued-recall task, and then they also encoded a list of 6 words using the imagery strategy followed by a cued-recall task. These two lists were also counterbalanced in order of presentation.

### 2.3 Data processing

Three memory scores were calculated per strategy condition: (1) a hit score (number of stems completed by a studied word and correctly judged as old), (2) a false alarm score (number of stems completed by an unstudied word and falsely judged as old), (3) a corrected hit rate corresponding to the hit score minus the false alarm score divided by the total number of targets. It should be noted that, in addition to counterbalancing the order of the strategies across blocks at the encoding stage, we also ensured that there were no effects of the order of the strategies or interactions with the variables of interest, and that it therefore made sense to group the three imagery strategy blocks on the one hand and the three repetition strategy blocks on the other (i.e., summing the memory scores for the three lists for each strategy).

To test our hypotheses, we conducted a mixed measures ANOVA with two factors: condition (Control vs. ABST, between) and strategy use (repetition vs. imagery, within). Openness was used as a linear covariate to test the interaction with condition and strategy use. In this analysis, we focused first on the main effect of condition and second on the higher order interaction involving condition. The dependent variable was the corrected hit rates.

## 3 Results

**Figure 2:**
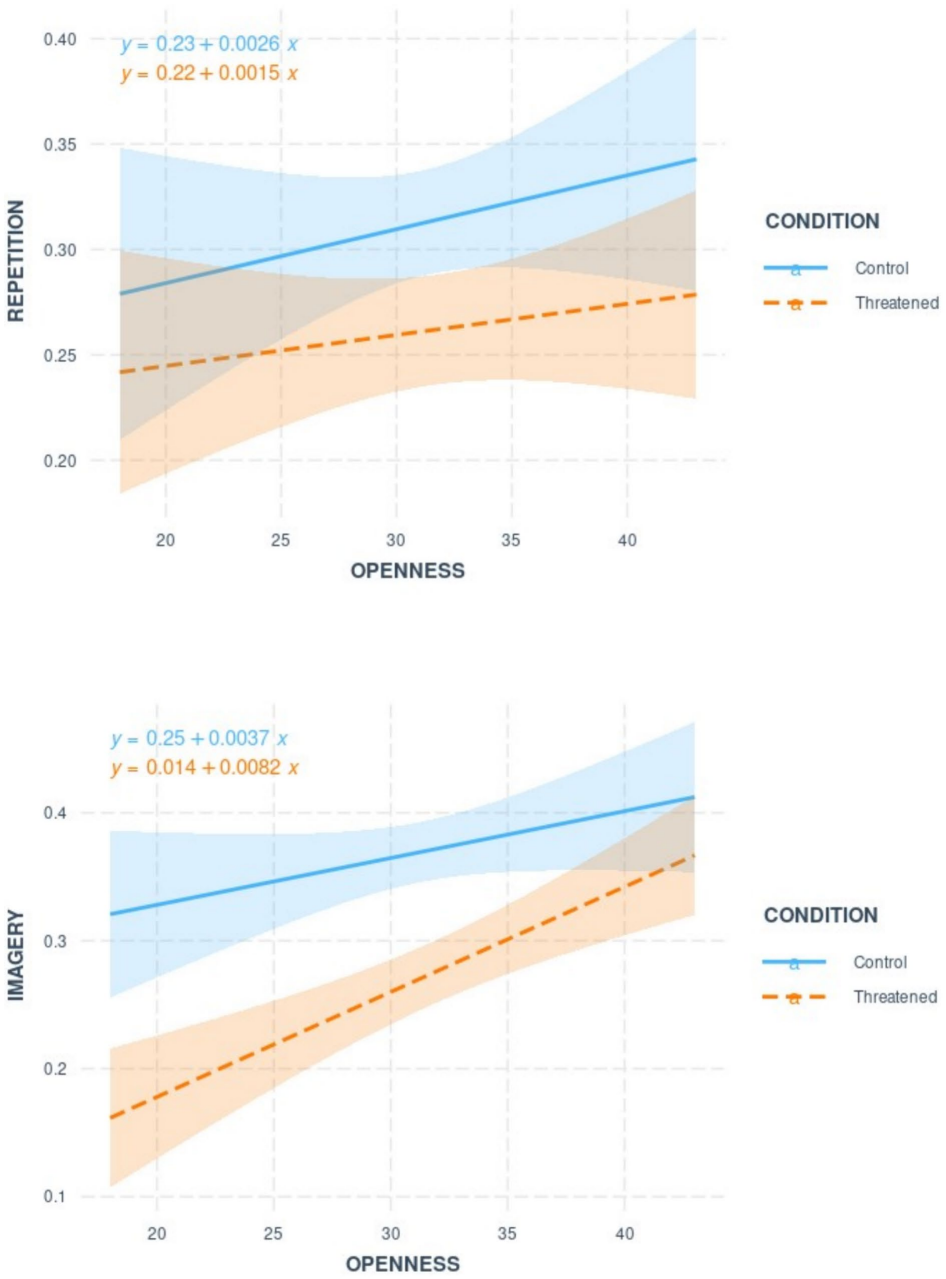
Regression slopes showing the relationship between openness scores and corrected hit rate as a function of the strategy and the condition.

The results of the ANOVA analysis (see Table 2) showed that the condition effect was significant when entered alone in the model, indicating that memory performance was significantly higher in the control group than in the threatened group. When openness was included in the model, the analyses showed that the effect of condition was no longer significant, but that it interacted with strategy, with the effect of condition being more important in the imagery condition than in the repetition condition, and with strategy and openness in a 3-way interaction. Regression analyses were performed to disentangle this high-level interaction (see Figure 2). These showed that participants in the ABST condition who scored higher on openness showed better memory performance when using the imagery strategy (*β* = 0.61, *p* = 0.000) compared to the repetition strategy (*β* = 0.12, *p* = 0.48). In the control condition, openness was not related to memory performance regardless of the strategy used (imagery: *β* = 0.25, *p* = 0.11; repetition: *β* = 0.18, *p* = 0.25).

**Table 2:** Results of ANOVA.

		Means (standard deviations)	
First model: condition factor alone	*F*(1, 73) = 19.22, *p* = 0.000, *η*^2^ = 0.20	Control: 0.34 (0.06)	Threatened: 0.26 (0.09)
Second model: all factors			
Condition	*F*(1, 71) = 2.30, *p* = 0.13, *η*^2^ = 0.03	Control: 0.34 (0.06)	Threatened: 26 (0.09)
Strategy	*F*(1, 71) = 4.24, p = 0.042, *η*^2^ = 0.05	Repetition: 0.28 (0.08)	Imagery: 0.32 (0.09)
Condition × Strategy	*F*(1, 71) = 6.56, *p* = 0.012, *η*^2^ = 0.08	Control: Repetition: 0.31 (0.07) Imagery: 0.37 (0.07)	Threatened: Repetition: 0.26 (0.08) Imagery: 0.27 (0.09)
Openness	*F*(1, 71) = 8.90, *p* = 0.003, *η*^2^ = 0.11		
Condition × Openness	*F*(1, 71) = 0.42, *p* = 0.51, *η*^2^ = 0.005		
Openness × Strategy	*F*(1, 71) = 8.27, *p* = 0.005, *η*^2^ = 0.10		
Condition × Openness × Strategy	*F*(1, 71) = 4.26, *p* = 0.042, *η*^2^ = 0.05		

## 4 Discussion

The aim of this research was to improve our understanding of the effect of ABST on episodic memory by exploring whether it affects the implementation of memory strategies at encoding. We also investigated the potential protective role of openness, hypothesizing that a high level of this personality trait would reduce the stereotype threat effect and help the implementation of a more resource-dependent memory strategy.

The results confirm previous findings that older participants recall fewer words when under stereotype threat (Lamont et al., 2015; Armstrong et al., 2017; Barber, 2017). As reported in the literature, imagery, by generating deep encoding, leads to better recall than repetition (Craik and Lockhart, 1972; Paivio and Csapo, 1973; Froger et al., 2012; Hertzog et al., 2012; Burger et al., 2017). We observed that threat mainly affected memory performance in the imagery condition, partly confirming the findings of Lemaire et al. (2018) who found a significant effect of ABST only on words encoded with imagery, with no effect on words encoded with the repetition, whereas we found an effect on both strategies, but to a greater extent for imagery. This discrepancy could be explained by differences in the experimental design, as we used repeated measures for strategy execution, whereas Lemaire et al. (2018) used independent groups. Moreover, we used several experimental blocks to obtain a number of trials for each strategy, which may have allowed an effect to emerge statistically even on the repetition strategy. In any case, ABST mainly effects the ability to implement the strategy that is most resource-demanding but also the most effective. Consequently, participants under threat did not benefit from the effect of imagery strategy, in contrast to those in the control group whose memory performance improved through use of the imagery strategy. When under threat, participants have to manage intrusive thoughts and stress; consequently, their cognitive resources are less available to perform a task, especially when this requires high cognitive resources, notably for older adults whose cognitive resources are reduced due to aging (Rabinowitz et al., 1982).

As expected, openness was associated with memory performance, the most open-minded individuals achieving the best performance. This result is in accordance with previous research investigating the role of openness in episodic memory performance (Soubelet and Salthouse, 2010; Simon et al., 2020; Karsazi et al., 2021; Martinez et al., 2021; Taconnat et al., 2022a). The beneficial effect of openness can be explained by the fact that individuals with high levels of openness tend to engage in a wide range of activities, providing strong cognitive stimulation that promotes the development of crystallized and fluid intelligence (Chamorro-Premuzic and Furnham, 2004; Soubelet and Salthouse, 2010; von Stumm et al., 2011); both these types of intelligence support memory functioning in aging (Bouazzaoui et al., 2013, 2014; Taconnat et al., 2022b). This result is also in accordance with recent study showing that high level of openness is associated with the preservation of the brain’s memory network and then with episodic memory performance and may be considered as a protective factor against aging (Stolz et al., 2023). As expected, we found that openness impacted differently strategies implementation, and that high levels of openness were positively associated with the recall of words encoded with an imagery strategy, but not with those encoded with a repetition strategy. Examining the role of openness on strategy use, Talpain and Soubelet (2020) found a positive association with both strategies, but that people with high levels of openness used imagery more than repetition. They also found that the relationship between openness and memory performance was mediated more by imagery than by repetition. Two major conclusions can be drawn from these findings. First, by facilitating efficient strategy use, openness provides executive support for memory functioning. This is in line with previous findings of a relationship between openness and executive functioning (DeYoung et al., 2005), and of the role of executive control in supporting episodic memory functioning and strategy use (Bouazzaoui et al., 2010, 2013, 2014; Bryan et al., 1999; Taconnat et al., 2006, 2007, 2022b, and see Lemaire, 2010 for a review). Secondly, the observation that openness was correlated only with the recall of words learned with the imagery strategy confirms previous studies and supports our hypothesis that openness is involved in the most effortful memory conditions (Talpain and Soubelet, 2020; Martinez et al., 2021; Taconnat et al., 2022a). By stimulating cognitive resources, openness acts as a cognitive reserve factor supporting memory performance, particularly in difficult tasks, by facilitating the use of a high resource-demanding strategy (Franchow et al., 2013; Luchetti et al., 2016; Jackson et al., 2020; Simon et al., 2020; Karsazi et al., 2021). The main and novel result of this study is that openness was only related to the efficiency of the imagery strategy in the ABST group. Being stereotyped as old makes older adults vulnerable, depleting their resources. However, high openness seems to be a protective factor, as it reduces the negative effects of ABST and helps to implement the more resource-dependent memory strategy. Our stereotype threat condition can thus be likened to the reading condition in the generation effect paradigm (Slamecka and Graf, 1978) and not performing a prior task in the prior-task-success paradigm (Taconnat et al., 2022a), which were considered as low environmental support conditions (Craik, 1986; Taconnat and Isingrini, 2004). In fact, the threat situation can also be considered as an experimental condition, which is very costly in terms of processing resources and requires more support to cope with the difficulty. Our results confirm that openness enhances memory performance and efficient strategy use in resource dependent conditions, such as when confronted by stereotype threat.

Future research could address the limitations of this study. The effect of stereotype threat on memory performance led us to believe that participants were stereotype threatened by the manipulation we used. However, it would be interesting to check whether participants actually felt threatened by questioning them at the end of the experiment, as other studies have done (Chasteen et al., 2005). Also, openness to experience is not the only personality trait associated with memory functioning. Taking a holistic view of personality, it would be interesting to test the role of other dimensions (and their possible interactions) in the relationship between memory, strategies and stereotype threat. Another research perspective would be to test our hypothesis using a memory task that is even more demanding task than cued recall. Indeed, several studies have used free recall, a task that requires important cognitive resources. The choice of task may have minimized the effects of stereotype threat, and also the relationship between strategies implementation and openness, even in the control group. Future research could also test the relationship between openness and strategy selection. Our study shows that openness affects the execution of memory strategies, but we did not test whether this is the case for strategy selection, which is also known to be affected by age-related stereotype threat. It would therefore be interesting to see whether openness plays a moderating role in the effect of stereotype threat on strategy selection. Finally, the literature on the moderators of the threat effect is now extensive and several hypotheses have emerged in recent years. In particular, there are psychosocial factors that may interact with personality, such as motivation and task commitment. Future studies could therefore investigate these relationships.

In sum, older adults under threat have difficulty using efficient strategies, resulting in impaired episodic memory performance. However, our results show that individual characteristics, such as a high level of openness, can counteract the negative effect of ABST by facilitating the implementation of efficient but resource-demanding strategies. Then, personality, and more specifically openness to experience, seems to be a variable to consider when assessing older people’s episodic memory, especially in a context that generates a sense of threat and stress. Further research is needed to replicate these results and also to clarify certain discrepancies observed in different studies, for example whether ABST affects the use of both repetition and imagery strategies, as found in our study, or only imagery (Lemaire et al., 2018), and whether openness is related to memory performance with both strategies (Talpain and Soubelet, 2020) or only with the imagery strategy (our study). These discrepancies could be due to differences in experimental design.

## Data availability statement

The data are available on the Open Science Framework depository here: https://osf.io/q6tjv/?view_only=6fa491a65d984b76a8261e22c6bae0df.

## Ethics statement

The studies involving humans were approved by Comité d’Ethique pour la Recherche, Tours-Poitiers. The studies were conducted in accordance with the local legislation and institutional requirements. The participants provided their written informed consent to participate in this study.

## Author contributions

BB: Writing – review & editing, Writing – original draft, Validation, Supervision, Software, Methodology, Investigation, Formal analysis, Data curation, Conceptualization. SF: Writing – review & editing, Validation, Methodology, Conceptualization. EA: Writing – review & editing, Investigation. LM: Writing – review & editing, Investigation. FP: Writing – review & editing, Methodology, Investigation. NK: Writing – review & editing, Investigation. TO: Writing – review & editing, Investigation. LT: Writing – review & editing, Validation, Project administration, Methodology, Funding acquisition, Conceptualization.
